# Risk of Cardiovascular Diseases in Diabetes Mellitus and Serum Concentration of Asymmetrical Dimethylarginine

**DOI:** 10.1155/2013/189430

**Published:** 2013-09-26

**Authors:** Seema L. Jawalekar, Aarti Karnik, Anil Bhutey

**Affiliations:** ^1^Department of Biochemistry, Sree Narayana Institute of Medical Sciences, Chalakka, Ernakulam, Kerala, India; ^2^Department of Biochemistry, ACPM Medical College, Dhule, Maharashtra, India; ^3^Department of Biochemistry, Panjabrao Deshmukh Medical College, Amravati, Maharashtra, India

## Abstract

*Introduction*. Asymmetric dimethylarginine (ADMA) is a nonselective nitric oxide (NO) synthase inhibitor associated with cardiovascular and metabolic disorders. ADMA plays an important role in the regulation of vascular tone by acting as an endogenous inhibitor of NO synthesis. *Objectives*. This study aimed to investigate ADMA with respect to diabetes and its clinical relevance as an independent predictor of CAD (Coronary Artery Disease). *Methodology*. The present case control study includes two hundred and forty patients selected randomly. Serum ADMA was analyzed by using enzyme immunoassay for the quantitative determination of endogenous ADMA, and serum nitric oxide was estimated by the method of Cortes. *Results*. Elevated NO level levels was a strong predictor and significantly (*t*: 9.86, *P* < 0.001) associated with occurrence of CAD. Increased ADMA level was found to be another strong predictor and associated significantly (*t*: 8.02, *P* < 0.001) with CAD. On intra group analysis, the relationship between ADMA and NO in diseased group, is significant negative correlation (*r* = −0.743). *P* (0.001) was found between ADMA and NO. *Conclusion*. ADMA level was found to be one of the strong predictors for CAD. ADMA is an emerging independent risk marker for future CVD (cardiovascular disease) events.

## 1. Introduction

Cardiovascular disease (CVD) is the major cause of morbidity and mortality in patients with diabetes mellitus (DM). Diabetes is at high risk for several cardiovascular disorders: coronary heart disease, stroke, peripheral arterial disease, cardiomyopathy, and congestive heart failure [1–3]. Endothelial dysfunction is a common feature in diabetic patients and may contribute to cardiovascular morbidity [4–6]. Mechanisms of diabetes-induced endothelial dysfunction include the production of prostanoid vasoconstrictors and the increased oxidative degradation of NO [7, 8]. Deficiency of NO increases vascular resistance and promotes atherogenesis [9]. 

Nitric oxide (NO) is a very active but short lived, a molecule that is released into the circulation from endothelial cells. It is a potent vasodilator that regulates vascular resistance and tissue blood flow. In addition, NO inhibits key processes of atherosclerosis, such as monocyte endo-thelial adhesion, platelet aggregation, and vascular smooth muscle cell proliferation. Hence, endothelial dysfunction due to reduced NO availability is an early step in the course of atherosclerotic vascular disease [10]. NO is synthesized by stereospecific oxidation of the terminal guanidino nitrogen of the amino acid, L-arginine, by the action of a family of NOS. Since NO has tremendous biological activity, its blood and/or tissue concentration must be kept within narrow limits in order to prevent harmful effects on cell activity. The synthesis of NO can be blocked, however, by inhibition of the NOS active site with guanidino-substituted analogs of L-arginine, such as asymmetrical dimethylarginine (ADMA) [10].

ADMA is a naturally occurring amino acid that has the interesting property of competitively inhibiting the activity of nitric oxide synthase (NOS). ADMA is produced by methylation of arginine residues in intracellular proteins via protein arginine *N*-methyltransferases (PRMT). 

After hydrolyzation of proteins, results into release of ADMA. ADMA is excreted in the urine, and, not surprisingly, there is an increase in plasma ADMA in patients with end-stage renal disease [11]. Parenthetically, patients with renal disease have an increased risk of cardiovascular morbidity and mortality, and in these patient's plasma ADMA concentrations carry prognostic information [12]. 

 The primary route of ADMA clearance, however, is by enzymatic degradation. Dimethylamine dimethylaminohydrolase (DDAH) converts ADMA to citrulline and dimethylamine. By regulating the plasma and tissue concentrations of ADMA, NOS activity is protected by DDAH [13].

Recently, it has been demonstrated that plasma levels of ADMA are elevated in patients with diabetes mellitus [7]. These findings suggest that the elevated ADMA in diabetes could contribute to accelerated atherosclerosis in this population. Further, as ADMA is mainly metabolized by dimethylarginine dimethylaminohydrolase (DDAH), it is conceivable that the inhibition of ADMA via upregulation of DDAH may be a novel therapeutic target for the prevention of CVD in patients with diabetes.

Under experimental conditions that lead to suboptimal L-arginine concentrations or to a relative deficiency of essential cofactors for NO synthase, the activity of this enzyme is “uncoupled.” This means that the oxidation of L-arginine to NO is not complete [14–16]. Normally, five electrons are being transferred in two steps of a coupled reduction-oxidation reaction by the two domains of NO synthase from molecular oxygen to L-arginine, resulting in the release of L-citrulline and NO.

ADMA is elevated to a level that can significantly inhibit NOS activity in individuals with hypercholesterolemia, hypertension, and hyperhomocysteinemia, tobacco exposure, and hyperglycemia [17–19] in each of these conditions; the elevation in ADMA level is a result of oxidative stress. Further oxidative stress impairs the ability of DDAH to metabolize ADMA [17, 20]. Hyperglycemia can elevate intracellular oxidative stress through multiple mechanisms [21, 22].

Under suboptimal conditions like those mentioned above, the electron flow within the two domains of NO synthase is disturbed, and molecular oxygen acts as an electron acceptor. This makes NO synthases a superoxide (O_2_^−^) radical-producing enzyme.

The endothelium-derived relaxation factor, nitric oxide, provides a unifying mechanism for the action of many cardiovascular disease risk factors. Nitric oxide cannot be measured directly, but the inhibitor of its formation and asymmetric dimethylarginine (ADMA) can be measured. The function of ADMA in modulating nitric oxide production has a significant impact on cardiovascular endothelial function.

## 2. Objectives

Asymmetric dimethylarginine (ADMA) is a nonselective nitric oxide (NO) synthase inhibitor associated with cardiovascular and metabolic disorders. This study aimed to investigate ADMA with respect to diabetes and its clinical relevance as an independent predictor of CAD.

## 3. Materials and Methods

The present case control study includes two hundred and forty patients, selected randomly. After careful clinical examination and confirmed diagnosis, they are further divided into three groups. Group I, patients presenting CVD (*n* = 80); Group II, patients with type II diabetes mellitus (*n* = 80); Group III, patients with type II DM with CVD; and a control group (*n* = 80). 

All patients selectedunderwent medical examination by a physician. A careful medical history was taken to obtain information about other diseases (particularly hypertension, coronary heart disease, myocardial infarction, stroke, peripheral vascular disease and endocrine disorders). Inclusion criteria, and patients clinically diagnosed having angiographically proven CVD with/without type II DM. Prevalence of diabetes was assessed either by fasting blood glucose level ≥126.0 mg/DL or HbA1C levels more than 6%. Exclusion criteria—patients with a history of prior coronary artery bypass grafting and patients with kidney and acute liver diseases were excluded.

Patients matched 80 subjects as control, and group II was randomly selected from patients attending diabetic OPD (Outpatient department). Controls have a negative history of CVD, Type II DM and had a normal resting ECG (Electrocardiogram).

Patients in group I and III were recruited from inpatient and outpatients departments of the hospital. The diagnosis of CVD was made on the basis of the clinical history (typical angina, history of myocardial infarction) and 12—lead standard ECG before subjecting them to coronary angiography. The presence of any stenosis >30% according to coronary angiography by visual assessment of coronary artery was included in the study. 

The study was approved by the ethical committee of our institute. Fully informed consent was obtained from patients. Blood samples were drawn into vacutainer tubes. Serum ADMA was analyzed by using enzyme immunoassay for the quantitative determination of endogenous ADMA, [23] and serum nitric oxide was estimated by the method of Cortes [24].

ADMA was analysed by enzyme linked immunoassay for the quantitative determination of endogenous asymmetric dimethylarginine in serum, manufactured by DLD diagnostics GMBH, which is based on principal, the competitive ADMA-ELISA uses the microtiter plate format. ADMA is bound to the solid phase of the microtiter plate. ADMA in the samples is acetylated and competes with solid phase bound ADMA for a fixed number of rabbit anti-ADMA antiserum binding sites. When the system is in equilibrium, free antigen and free antigen antiserum complexes are removed by washing. The antibody bounded to the solid phase ADMA is detected by the anti-rabbit/peroxidase. The substrate TMB/peroxidase reaction is monitored at 450 nm. The amount of antibody bound to the solid phase ADMA is inversely proportional to the ADMA concentration of the sample. 

Statistical analysis was carried by student's *t*-test and chi-square test. Results were expressed as mean ± SD. Probability values of 0.05 were considered statistically significant. Associations between two categorical variables were tested by means of the *χ* ^2^ or Fisher exact tests. All baseline characteristics, presented in Table 1, were evaluated as potential confounders, using univariate Cox regression analysis. All tests were two tailed, and *P* < 0.05 was considered statistically significant. Statistical analysis was performed with SPSS statistical software (V.13.0).

## 4. Results 

Patients who had no diabetes mellitus and cardiovascular disease were grouped as control; diabetes with CVD and nondiabetes with CVD are categorized as diseased group and others are categorized as nondiseased group.

Baseline characteristics of the participants are presented in Table 1. The overall mean age for control was 47.0 years, and diseased group was 56.0 years; 74.7% were men. Diabetes with CVD had higher systolic blood pressure and hyperlipidemia than normal healthy subjects.  Table 2 shows that type II diabetes with CAD patients have significantly higher ADMA concentration than normal healthy subjects and other study groups *P* < 0.001. NO concentration was lower lowest 26.71 ± 3.41 in DM with CAD patients than the other study groups and normal healthy subjects.

In a multiple logistic regression analysis adjusting for hypertension, hypercholesterolemia, low HDL cholesterol and diabetes or fasting glucose, ADMA remained a strong and independent predictor for the presence of CAD. Elevated No level levels was a strong predictor and significantly (*t*: 9.86, *P* < 0.001) associated with occurrence of CAD. Increased ADMA level was found to be another strong predictor and associated significantly (*t*: 8.02, *P* < 0.001) with CAD. 

The univariate logistic regression analysis for risk factors versus CVD (as a dependent variable) was done to assess relative risk of development of CVD with each risk factor in the entire study population that includes diseased and nondiseased subjects. Age, family history, obesity, and SBP and DBP are highly significant and associated with CAD pathogenesis (Tables 2, 3, and 4).

NO cut-off level was 30 *μ*mol/L. All study groups indicate presence of disease. Our data supports NO which is also one of the independent risk factors and significantly (*χ* ^2^:  40.65, *P* < 0.001) associated with pathogenesis of CAD. The cut-off level for ADMA was 0.60 *μ*mol/L (*χ* ^2^:   36.46, *P* < 0.001), which is another independent risk factor for CAD.

Diagnostic validity was done for assessing a good and better novel predictor for CAD. Sensitivity (presence of the disease if ≤30 *μ*mol/L) of NO was found to be 53.20% and specificity (absence of the disease if ≤30 *μ*mol/L) was 95.40%. PPN (positive predictor value) of NO was 90.0%, and NPV (negative predictor value) was 68.10%. Overall accuracy or diagnostic efficiency of NO was found to be 76.0%. Sensitivity (presence of the disease if ≤30 *μ*mol/L) of ADMA was found to be 74.40% and specificity (absence of the disease if ≤30 *μ*mol/L) was 82.80%. PPN (positive predictor value) of ADMA was 91.10%, and NPV (negative predictor value) was 68.10%. Overall accuracy or diagnostic efficiency of NO was found to be 79.20%. Odds ratios for NO were 19.10 and 7.11 for ADMA (Table 5).

**Figure 1:**
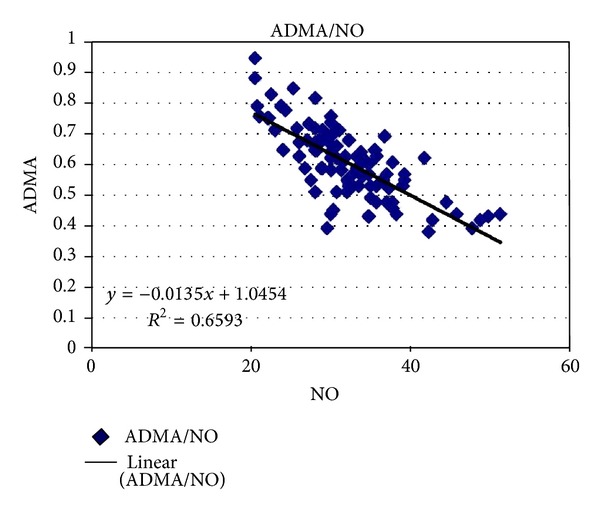
Correlation between ADMA and NO in diseased group.

Figure 1 reports the relationship between ADMA and NO in diseased group, on intra group analysis there is significant negative correlation (*r* = −0.743); *P* (0.001) was found between ADMA and NO. It suggests that increase in ADMA associated with decrease is in NO activity or concentration. 

## 5. Discussion

Associations between increased levels of ADMA and many cardiovascular risk factors such as age, hypertension, diabetes, insulin resistance, hypercholesterolemia, hypertriglyceridemia, and hyperhomocysteinemia have been documented [25–27]. Evidence for a causal relationship between increased ADMA levels and endothelial vasodilator dysfunction has been demonstrated in many of these conditions. Hyperglycaemia is associated with endothelial dysfunction both *in vivo* [28] and *in vitro* [29], therefore, endothelial dysfunction is an early feature in the development of vascular complications in people with diabetes [30]. Patients with diabetes have an adverse cardiovascular risk profile. Elevated ADMA concentration has been described in patients with type 2 And type 1 diabetes [25]. Hyperglycemia probably may increase ADMA concentration. One invite study demonstrated that elevated glucose levels are capable of inhibiting DDAH activity in cultured endothelial cells [31]. Clinical investigations in patients also indicate that ADMA is directly related to blood glucose levels [25, 32]. As demonstrated in a recent study, strict glycemic control may exert antiatherogenic effects by reducing ADMA levels in patients with type 2 diabetes [33]. Consistent with these studies, the present study provide evidence that elevated serum ADMA and reduced NO independently associated with cardiovascular risk in diabetes patients with coronary artery disease. ADMA might act as pathophysiologically relevant factor for diabetes associated complications. Nevertheless, hyperglycemia remains a major cause for both increased ADMA and the development of diabetic complications which makes the interpretation of the data more complex.

 ADMA was that atherosclerosis is marked by impaired vasodilation in response to normal physiological stimuli, and the impairment can be relieved by intravenous L-arginine [34]. A number of standard heart disease risk factors (smoking, hypertension, hyperlipidemias) are related to vasodilatory impairment, but the strongest correlation may be the ADMA in plasma [35]. Elaboration of endothelium-derived nitric oxide affects the behaviour of circulating T lymphocytes and monocytes. Mononuclear cell adhesiveness is inversely correlated with the plasma ADMA, and the effect is reversed by restoration of the ratio-to-control levels with oral administration of 14–21 grams of arginine [36]. These changes lead to lower rates of atherosclerotic plaque formation and lower risk of heart disease. In addition, the association of elevated LDL cholesterol with increased risk of heart disease may be a covariable in the oxidative activation of ADMA synthesis. Oxidized LDL particles stimulate the expression of enzymes that generate ADMA [37].

ADMA may contribute to abnormal blood flow responses and to atherogenesis in type 2 diabetics [38]. In hypertensives, the urinary excretion rate of nitrite plus nitrate (NOx), an index of endogenous NO production, was lowered from 77 to 56 micromol/mmol creatinine, while plasma levels of ADMA were increased from 1.1 to 2.4, indicating ADMA inhibition of NO production [39]. ADMA plays an important role in the pathogenesis of hypertension associated with the experimental focal and segmental glomerulosclerosis [40].

ADMA is released after posttranslational methylation from nuclear proteins involved in RNA processing and transcriptional control. The enzyme protein arginine methyltransferase type I (PRMT I) forms ADMA, whereas PRMT II forms symmetric dimethylarginine (SDMA) (i.e., the stereoisomer of ADMA with no known effect on NOS). Other data suggests that human endothelial cell PRMT activity is upregulated by LDL cholesterol, due in part to the enhanced gene expression of PRMTs [37]. Thus, we find ADMA intermeshed with both cholesterol, and homocysteine cardiovascular risk factors. Hyperglycemia is another factor related to ADMA. Human endothelial cells exposed to high glucose concentrations show impairment of DDAH and accumulation of ADMA that may contribute to endothelial vasodilator dysfunction in diabetes mellitus [31]. Improved glycemic control in patients with type 2 diabetes results in lowering of plasma ADMA levels.

As ADMA directly impairs the physiological NO-dependent functions of the endothelial lining, its primary pathophysiological mechanism of action is different from all other known risk factors like hypertension (pressure overload of the arterial wall and reduced arterial elasticity), hypercholesterolemia (uptake of (oxidized) LDL into the intimal layer, generation of foam cells, and local inflammation), smoking (induction and potentiation of oxidative damage of cellular structures within the arterial wall). Accordingly, it can be expected that the deleterious effects of ADMA are independent of other risk factors and add to their effects. 

In our study, ADMA level was found to be one of the strong predictors for CAD. Another group of investigators from The Netherlands studied the survival of patients undergoing intensive care unit treatment and aimed to identify novel risk factors for survival during ICU treatment [41]. Among all biochemical markers of organ function and disease risk that were measured in this study, ADMA was the factor with the highest predictive power. Patients with elevated ADMA levels had a 17-fold increased risk of fatality during ICU treatment [41]. 

Understanding of the pathophysiological role of ADMA for the development of cardiovascular diseases, this molecule has become a novel goal for therapeutic intervention. ADMA plays an important role in the regulation of vascular tone by acting as an endogenous inhibitor of NO synthesis. By inhibiting NO synthesis, plasma ADMA may reduce vascular compliance, increase vascular resistance, and limit blood flow. Furthermore, plasma ADMA may promote atherogenesis as it opposes the vasoprotective effects of NO. Thus, elevations in plasma ADMA may accelerate the progression of atherosclerosis and increase the risk of cardiovascular events.

In conclusion, ADMA is an emerging independent risk marker for future CVD events. The clinical acceptance of this parameter will depend on the availability of a therapy to directly decrease ADMA, which could then confirm the role of ADMA as a causal risk factor. Further studies are warranted in patients with diabetes, especially regarding the possible effects of ADMA on diabetes associated complications.

## Figures and Tables

**Table 1:** Baseline characteristics of the study population.

	Control	Diseased	*P* value
Age (years)	47.29 ± 10.21	56.21 ± 6.73	<0.10
Family history	13	24	—
Smoker	4	22	—
BMI	21 (>25)	31 (>25)	—
BP systolic (mm/Hg)	122.84 ± 3.45	132.53 ± 7.62	<0.01
BP diastolic (mm/Hg)	80.14 ± 5.53	86.23 ± 6.54	<0.20
F BSL (mg/dL)	95.60 ± 18.38	132.80 ± 32	<0.01
HbA1C	6.0 ± 1.27	7.32 ± 2.46	<0.50
Total cholesterol	159.86 ± 26.98	282.36 ± 28	<0.01
HDL (mg/dL)	44.21 ± 8.35	28.67 ± 8.89	<0.01
LDL (mg/dL)	102.02 ± 25.78	159.69 ± 29.65	<0.05
VLDL (mg/dL)	32.98 ± 10.43	45.31 ± 14.30	<0.02
TG (mg/dL)	172.67 ± 56.87	227.38 ± 68.79	<0.01

**Table 2:** Risk factors.

Variables	Control	Group I	Group II	Group III	*F*	*P* value
NO *μ*mol/L	52.36 ± 6.09	32.01 ± 5.005	43 ± 8.62	26.71 ± 3.41	120.8	0.001
ADMA *μ*mol/L	0.36 ± 0.30	0.66 ± 0.76	0.49 ± 0.28	0.78 ± 0.83	7.633	0.001

**Table 3:** Parameters taking CAD as dependent variable.

Variable	Diseased group	Nondiseased group	*χ* ^2^	*P* value
NO <30.00 *μ*mol/L	52.6 ± 7.79 51%	27.38 ± 4.06 6%	80.44	<0.0001
ADMA >0.60 *μ*mol/L	0.40 ± 0.65 72%	0.69 ± 0.85	135.1	<0.0001

**Table 4:** Univariate analysis of cofounding factors.

Variable	Particular	Diseased group	Nondiseased group	95% CI	*P* value
NO	<30.0 *μ*mol/L	54.34	6.2	38.18–42.67	<0.0001
ADMA	>0.60 *μ*mol/L	0.81	0.63	0.62–1.16	<0.0001

**Table 5:** Prognostic value of novel markers.

Variable	Specificity	Sensitivity	Positive predictive value	Negative predictive value	Diagnostic accuracy	Odd ratio
NO	53.20%	95.40%	90.0%	68.10%	76.0%	19.10
ADMA	74.40%	82.80%	91.10%	79.20%	84.00%	7.11

## Abbreviations

ADMA: Asymmetric dimethylarginine
CAD: Coronary artery disease
CVD: Cardiovascular disease
DM: Diabetes mellitus
DDAH: Dimethylamine dimethylaminohydrolase
ECG: Electrocardiogram
NO: Nitric oxide
NOS: Nitric oxide synthase
OPD: Outpatient department
PRMT: Protein arginine *N*-methyltransferases.
